# Equine seroprevalence of West Nile virus antibodies in the UK in 2019

**DOI:** 10.1186/s13071-020-04481-9

**Published:** 2020-11-26

**Authors:** Arran J. Folly, Elisabeth S. L. Waller, Fiona McCracken, Lorraine M. McElhinney, Helen Roberts, Nicholas Johnson

**Affiliations:** 1 Virology Department, Animal and Plant Health Agency, Woodham Lane, Surrey, UK; 2 Institute of Infection and Global Health, University of Liverpool, Liverpool, UK; 3 Exotic Disease Control Team, Defra, 17 Smith Square, London, UK; 4 Faculty of Health and Medicine, University of Surrey, Guildford, UK

**Keywords:** *Flaviviridae*, Equidae, Emerging diseases, Viraemia, Enzyme-linked immunosorbent assay, Serosurveillance

## Abstract

**Background:**

West Nile virus (WNV) is a single-stranded RNA virus that can cause neurological disease in both humans and horses. Due to the movement of competent vectors and viraemic hosts, WNV has repeatedly emerged globally and more recently in western Europe. Within the UK, WNV is a notifiable disease in horses, and vaccines against the virus are commercially available. However, there has been no investigation into the seroprevalence of WNV in the UK equine population to determine the extent of vaccination or to provide evidence of recent infection.

**Methods:**

Equine serum samples were obtained from the Animal and Plant Health Agency’s equine testing service between August and November 2019. A total of 988 serum samples were selected for horses resident in South East England. WNV seroprevalence was determined using two enzyme-linked immunosorbent assays (ELISAs) to detect total flavivirus antibodies and WNV-specific immunoglobulin M (IgM) antibodies. Positive IgM results were investigated by contacting the submitting veterinarian to establish the clinical history or evidence of prior vaccination of the horses in question.

**Results:**

Within the cohort, 274 samples tested positive for flavivirus antibodies, of which two subsequently tested positive for WNV-specific IgM antibodies. The follow-up investigation established that both horses had been vaccinated prior to serum samples being drawn, which resulted in an IgM-positive response. All the samples that tested positive by competition ELISA were from horses set to be exported to countries where WNV is endemic. Consequently, the positive results were likely due to previous vaccination. In contrast, 714 samples were seronegative, indicating that the majority of the UK equine population may be susceptible to WNV infection.

**Conclusions:**

There was no evidence for cryptic WNV infection in a cohort of horses sampled in England in 2019. All IgM-seropositive cases were due to vaccination; this should be noted for future epidemiological surveys in the event of a disease outbreak, as it is not possible to distinguish vaccinated from infected horses without knowledge of their clinical histories.
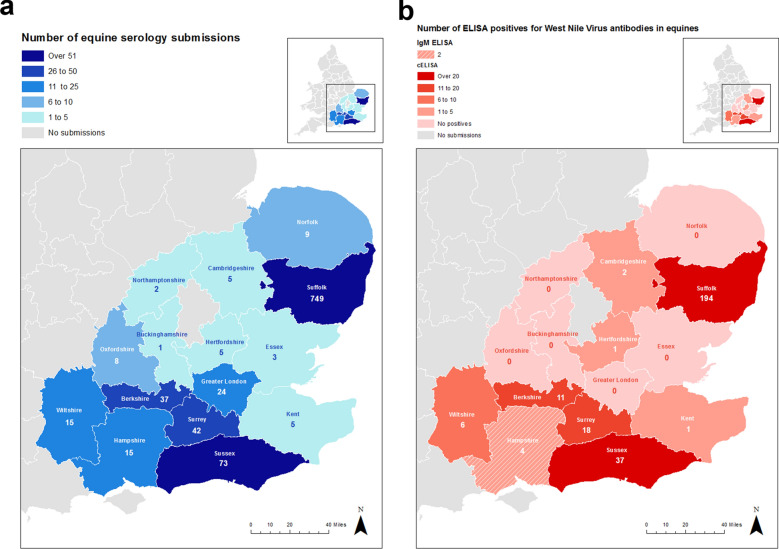

## Introduction

West Nile virus (WNV) (genus *Flavivirus*, family *Flaviviridae*) is the causative agent of West Nile fever in humans and encephalitis in both humans and horses [1, 2]. It is a single-stranded RNA virus which is maintained in a natural enzootic cycle between mosquito vectors and avian reservoirs. However, several competent mosquito WNV vectors are not host specific and this can result in transmission to and disease in both humans and equines. Consequently, the emergence of WNV can have a major impact on both public health and animal welfare.

First identified in Uganda in 1937, WNV is now found in many regions of the world; it occurs seasonally in countries of the Mediterranean Basin, and recent incursions across Germany have been reported [3]. Evolutionary analysis of the WNV genome suggests that the virus was introduced into Germany in 2016, but was not detected there until 2018. Critically, early detection of the emergence and transmission of a virus enables public health and veterinary services to plan for potential cases and helps to achieve more general epidemiological monitoring. One method for the early detection of WNV is the serosurveillance of sentinel horses, as reported from Spain [4], Germany [5] and Africa [6], for example. However, confounding factors in serosurveillance studies are the use of vaccination and potential serum cross-reactivity with related viruses [7].

Three vaccines have been licenced for use in equines for WNV in Europe [8], and discrimination between vaccinated and infected horses can be complex [9]. The UK is currently free of indigenously acquired WNV infection, with occasional human cases that are attributed to infection abroad [10]. Nevertheless, given that species of mosquito from the UK are competent vectors of WNV [11], and that there is migration of potentially viraemic birds from northern Europe, it is becoming increasingly feasible that WNV could emerge in UK mosquito populations [12]. Despite these concerns, there has been no investigation into the seroprevalence of WNV in equines in the UK. Critically, this approach could be used to detect cryptic WNV infections, inform future disease intervention strategies and assist the development of epidemiological modelling. Here we use a combination of total antibody and immunoglobulin M (IgM; the first antibody to be produced in vertebrates in response to antigen exposure)-specific enzyme-linked immunosorbent assays (ELISAs; a plate-based technique used here for detecting and quantifying antibodies in host sera) to elucidate the seroprevalence of WNV antibodies in a cohort of equines resident in South East England during the vector active season of 2019.

## Methods

All serum samples used in this investigation were obtained from test submissions to the Animal and Plant Health Agency (APHA) equine testing service. Before being entered into the study, all serum samples had to meet three predefined criteria. First, horses must have been resident in South East England at the time of sampling (Additional file 1: Table S1), thus excluding potential imports from WNV endemic areas. This restricted the cohort to horses from a region at greater risk of WNV introduction due to more abundant populations of vectors and proximity to the near continent. Secondly, blood samples must have been taken during the summer and autumn months of 2019 to coincide with the period of vector activity in the UK. Finally, the sample must have been maintained within a cold chain (− 20 °C) before and after tests were carried out. Between August and November 2019, a total of 993 horse sera samples were submitted and 988 unique sera samples were selected comprising those submitted for either export (*n* = 515) or diagnosis (*n* = 473) that met all the criteria described above (Additional file 1: Table S1). Of the sera submitted for diagnosis, the tests requested were dourine (*n* = 343), equine infectious anaemia (*n* = 446), equine viral arteritis (*n* = 134), and glanders (*n* = 335); please note that multiple tests were requested for some of the sera.

To detect the presence of WNV antibodies, two commercially available (IDVet, Grabels, France) enzyme-linked immunosorbent assays (ELISA) kits were used in accordance with the manufacturer’s guidelines. In the first instance, sera were heat inactivated at 56 °C for 30 min and subjected to a competition ELISA (cELISA) that can detect the presence of total flavivirus antibodies. This test can indicate a historic or recent infection or vaccination. Following this initial screen, any samples that tested positive using the cELISA were then rescreened using an IgM-specific ELISA, which can detect a recent infection or recent vaccination. An assessment of IgM-seropositive horse samples involved contacting the submitting veterinarian to obtain the horse’s clinical status, to exclude recent infection, and to review its WNV vaccination history. All graphical outputs were undertaken in ArcGIS (v10.2.2) and regional differences in antibody prevalence were compared using a Chi-square test.

## Results

**Fig. 1:**
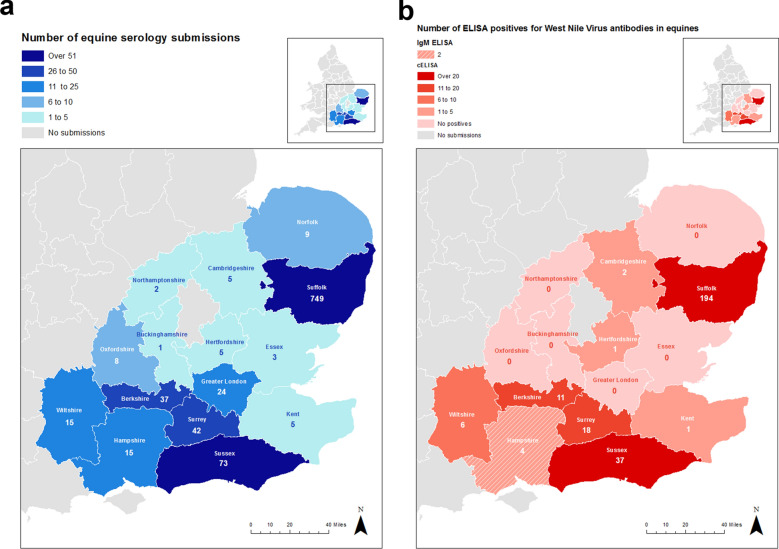
Choropleth map of England and Wales showing **a** total submission locations for sera that met our testing criteria (*n* = 993; NB due to duplicated submissions, only 988 sera were eligible for testing), and **b** enzyme-linked immunosorbent assays (ELISA)-positive locations for South East England. The location of equine samples that were competition ELISA positives can also act as a proxy to provide location data for West Nile virus-vaccinated horses

Of the 988 sera samples, 274 tested positive for flavivirus antibodies by cELISA (Fig. 1) giving a seroprevalence of 27.7% (95% CI 24.9–30.5%) and highlighting that 72.3% (95% CI 69.5–75.1%) of the cohort were seronegative and therefore potentially susceptible to WNV infection. Two of the positive cELISA samples also tested positive by the IgM ELISA. Post-test investigation indicated that all the horses which were positive for flavivirus antibodies by cELISA were healthy, that they were submitted for testing prior to export from the UK, and that they were likely vaccinated as they were travelling to areas where WNV is endemic. In addition, the two horses that tested positive for IgM antibodies had received a WNV vaccination 7 days prior to blood being drawn. Consequently, there was no evidence for cryptic WNV transmission, or for a historic emergence event in this equine cohort.

It is important to note that while Suffolk accounted for a large proportion of our samples (75.8%) (Fig. 1), our detected antibody response is dependent on region as there was a significant difference between the proportion of positive samples from other regions of South East England when compared to Suffolk, with Suffolk having a lower antibody prevalence (*χ*^2^ = 5.18, *P* = 0.02).

## Discussion

Here we show that from our cohort of equines, which were resident in South East England in 2019, there is currently no evidence for cryptic WNV infection or WNV emergence in this region of the UK. While some of the horses from our cohort were vaccinated against WNV, the majority were not. To our knowledge, this is the first equine serosurvey for WNV seroprevalence to be undertaken in the UK, and we believe that our results can be used as a baseline from which future WNV serosurveillance or outbreak investigations can be mapped.

The emergence of WNV has been seen across southern Europe, with repeated seasonal outbreaks in countries of the Mediterranean Basin, and most recently in Germany [3]. The ability of WNV to emerge is likely facilitated by the movement of viraemic birds, and its presence is usually detected following infection in dead-end hosts, such as horses or humans, in the absence of intensive wildlife surveillance. However, as most WNV infections in horses are asymptomatic or consist of a mild fever, cases can be missed when using syndromic surveillance alone. In surveys from European and African countries, WNV IgM antibodies have been used to detect recent infection in dead-end hosts, such as horses [4, 6]. Our dataset contains a comparable number of sentinel horses from a region of the UK that is hypothesised to be at higher risk of WNV emergence as it is considered to be at higher risk for the introduction of other arthropod-borne viruses such as bluetongue virus [13]. The results of this serosurvey, when combined with annual surveillance that has not detected WNV RNA in avian samples from the UK ([14], plus ongoing unpublished APHA data), indicate that, for South East England, there is no evidence for cryptic transmission of WNV during the vector active season of 2019.

The estimated UK equine population is 847,000, and of these horses the majority are thought to be for recreational use [15]. Partly as a result of the target geographical region used in this study, which included Suffolk, an area of the UK with a high density of sports stables, a significant proportion of the horses were involved in racing and polo, two global sports that require international travel and protection against exposure to exotic diseases that are not present in the UK. Consequently, it is likely that these horses regularly travel to countries where WNV is endemic, and are vaccinated against this disease as a precautionary measure. However, for almost half of the export horses from our cohort no flavivirus antibody was detectable, yet some of these animals had travelled to WNV endemic areas and thus could have been exposed to infection before returning to the UK, as occurred in a horse that returned from Cyprus in 2013 [16]. In addition, total antibody ELISAs may be susceptible to cross-reactivity resulting from host infection with related viruses; indeed, one serosurvey suggested that WNV seropositivity can be confounded with tick-borne encephalitis virus (TBEV) infection [5]. Until recently, the UK was thought to be free of TBEV, but it has recently been detected in ticks from southern England [17]. The impact of this on future WNV serosurveillance in UK horses is unclear and warrants further investigation. Consequently, differentiating vaccination, antigenic cross-reactivity and prior infection with WNV in equine populations is challenging. To overcome this, WNV serosurveillance studies have instead focused on IgM-specific ELISA results as these may be more accurate in ascertaining WNV infection and recent transmission [18]. Whilst this does not entirely remove the possibility that disease prevalence data may be misinterpreted, as evidenced by the two horses that tested positive for WNV IgM antibodies in this study, it can substantially reduce the likelihood of vaccine-associated IgM detection or infection-mediated cross-reactivity. Regarding the two IgM-positive horses, the clinical history was obtained for both, which confirmed that 7 days prior to blood samples being drawn WNV vaccinations had been administered.

Whilst we found no evidence of active or previous WNV infection in the sera studied, the proportion of horses that tested seropositive in our investigation highlights that understanding background WNV vaccination prevalence is important for interpreting results of serosurveillance within equine populations. Indeed, most horses from our cohort had no WNV-specific antibodies, and thus would be considered a susceptible population should WNV be introduced into the UK in the future.

## Conclusions

WNV is an emerging arbovirus in Europe. To date there has been no detection of this virus in the UK. Using flavivirus- and WNV-specific ELISAs, no evidence of cryptic WNV transmission was detected in a cohort of horses sampled in England during the vector active season of 2019. Two sera samples did test positive for WNV IgM antibodies. However, following review of the horses’ clinical records, the positive results were attributed to recent WNV vaccination. Consequently, vaccination prevalence may confound the interpretation of future serosurveys.

## Supplementary information

**Additional file 1: Table S1.** Summary of location data for equine submissions that met our criteria for testing. The reason for submission has been provided by the submitting veterinarian.

## Data Availability

The dataset supporting the conclusions of this article is available from the Figshare repository, under the title* Equine Seroprevalence for West Nile virus Antibodies in the United Kingdom, 2019*. https://doi.org/10.6084/m9.figshare.13020542.

## Abbreviations

ELISA: Enzyme-linked immunosorbent assay
IgM: Immunoglobulin M
TBEV: Tick-borne encephalitis virus
WNV: West Nile virus
