# Supplementary material for: Equine seroprevalence of West Nile virus antibodies in the UK in 2019
Source: Parasit Vectors. 2020 Nov 26;13:596. doi: 10.1186/s13071-020-04481-9 (PMC7690108; doi:10.1186/s13071-020-04481-9)
Supplement: Supplementary file 1 — Additional file 1: Table S1. Summary of location data for equine submissions that met our criteria for testing. The reason for submission has been provided by the submitting veterinarian. [file 13071_2020_4481_MOESM1_ESM.docx]

**Additional file 1: Table S1**. Summary of location data for equine submissions that met our criteria for testing. Reason for submission has been provided by the submitting veterinarian.

| County | Reason for submission | | Total |
| --- | --- | --- | --- |
|  | **Export** | **Diagnostic test** |  |
| Berkshire | 20 | 17 | 37 |
| Buckinghamshire | - | 1 | 1 |
| Cambridgeshire | 2 | 3 | 5 |
| Essex | 1 | 2 | 3 |
| Hampshire | 4 | 11 | 15 |
| Kent | 2 | 3 | 5 |
| London | - | 24 | 24 |
| Norfolk | - | 9 | 9 |
| Northamptonshire | 1 | 1 | 2 |
| Oxfordshire | - | 8 | 8 |
| Suffolk | 406 | 343 | 749 |
| Surrey | 27 | 15 | 42 |
| Sussex | 48 | 25 | 73 |
| Wiltshire | 4 | 11 | 15 |
| Total: | **515** | **473** | **988** |
